# Effect of Nitrogen on Precipitate Characteristics and Pitting Resistance of Martensitic Stainless Steel

**DOI:** 10.3390/ma17153817

**Published:** 2024-08-02

**Authors:** Hui Xu, Jinbin Wang, Yugui Li, Bin Wang, Huaying Li, Guangming Liu

**Affiliations:** 1 School of Materials Science and Engineering, Taiyuan University of Science and Technology, Taiyuan 030024, China; xuhui_tyust@163.com (H.X.); 5425wb@163.com (B.W.); huayne@163.com (H.L.); liugm@tyust.edu.cn (G.L.); 2 State Key Laboratory of Rolling and Automation, Northeastern University, Shenyang 110819, China; wjb0226@sina.com; 3 School of Mechanical Engineering, Taiyuan University of Science and Technology, Taiyuan 030024, China; 4 State Key Laboratory of Advanced Stainless Steel Taiyuan Iron and Steel (Group) Co., Ltd., Taiyuan 030003, China

**Keywords:** martensitic stainless steel, nitrogen, precipitation, passive films, pitting resistance

## Abstract

High-carbon–chromium martensitic stainless steel (MSS) is widely used in many fields due to its excellent mechanical properties, while the coarse eutectic carbide in MSS deteriorates corrosion resistance. In this work, nitrogen was added to the MSS to improve corrosion resistance. The effects of nitrogen on the microstructure and corrosion resistance of MSS were systematically studied. The results showed that the addition of nitrogen promoted the development of Cr_2_N and reversed austenite, effectively inhibiting the formation of δ-ferrite. Therefore, the durability of the passivation film was improved, the passivation zone was expanded, and the susceptibility to metastable pitting was decreased. As a consequence, nearly two orders of magnitude have been achieved in the pitting potential (E_pit_) of MSS containing nitrogen, and the polarization resistance value (R_p_) has gone up from 4.05 kΩ·cm^2^ to 1.24 × 10^2^ kΩ·cm^2^. This means that in a corrosive environment, nitrogen-treated MSS stainless steel is less likely to form pitting pits, which further extends the service life of the material.

## 1. Introduction

High-carbon–chromium martensitic stainless steel (MSS), as an important bearing material, finds widespread application in fields like marine vessels, aerospace, petrochemicals, and the nuclear industry, due to its high hardness and corrosion resistance [1,2]. However, the precipitation of coarse eutectic carbides reduces fatigue life and corrosion resistance [3,4]. As science, technology, and industry advance, there is a growing demand for materials with specific properties, especially in harsh conditions such as highly corrosive environments. This presents significant challenges for the microstructure and corrosion resistance of martensitic stainless steel. Therefore, research into new alloying methods to enhance the microstructure and properties of martensitic stainless steel has become a key focus in materials science.

Nitrogen (N), as a strong austenitic-forming element, is widely used in the steel alloying process [5,6,7]. Adding nitrogen to steel significantly impacts the microstructure of retained austenite, martensite shape, grain size, and precipitates [8,9,10]. It has been discovered that in 9Cr18Mo steel, substituting some of the C with N can decrease the quantity and size of eutectic carbides. As a result, the mesh eutectic carbides gradually vanish [11]. In a study by Li et al. [12], it was observed that increasing the nitrogen content in 4Cr13 steel reduced the precipitation of primary carbides and contributed to the refinement of dendrites.

Recently, researchers have revealed that adding nitrogen to steel enhances the interaction between N and Cr, resulting in the formation of fine nitrides and carbonitrides that disperse throughout the matrix [13]. This reduces the Cr that combines with C to form carbides, the carbon–chromium element segregation is reduced, the chance of large-grained carbide formation is reduced, and the carbide size is significantly refined [14,15].

In addition, although the thin (nanoscale) oxide layers naturally formed on the surface of stainless steel are effective in slowing corrosion, they are prone to localized breakdown, which accelerates the dissolution process of the underlying metal [16]. The presence of nitrogen can minimize defect density, increase the thickness of the passivation film, promote the enrichment of Cr in the passivation film, reduce metastable pitting sensitivity, and improve the ability of repassivation [17,18,19].

Leda et al. [20] reported that in 0.7C-13Cr martensitic stainless steel, the partial replacement of carbon with nitrogen (0.2 wt.%) can decrease pits. Shimada et al. [21] pointed out that when the performance of 0.65C-16Cr martensitic stainless steel is optimized, its nitrogen content should be controlled at 0.25 wt.%. Moreover, Qi et al. [22] showed that the corrosion resistance of nitrogenous MSS was excellent, but it did not show a monotonous trend with the increase in nitrogen content. Therefore, the action mechanism of nitrogen on MSS is complicated, and limited reports have been made to explain the effect of N on the microstructure evolution and the corrosion resistance of MSS. Thus, the effect of nitrogen on the corrosion resistance and microstructure evolution of MSSs is worthy to be systematically studied, which would provide valuable insights for the design and optimization of MSSs to adapt to industrial applications.

The work thoroughly investigates the nitrogen form in MSS and discusses its detailed impact on microstructure and electrochemical behavior. It also uncovers the specific mechanism through which nitrogen influences the microstructure and pitting resistance of MSS. The systematic analysis and experimental verification presented in this study provide an essential theoretical basis and practical guidance for understanding the role of nitrogen in MSS.

## 2. Materials and Methods

### 2.1. Materials Preparation

**Figure 1:**
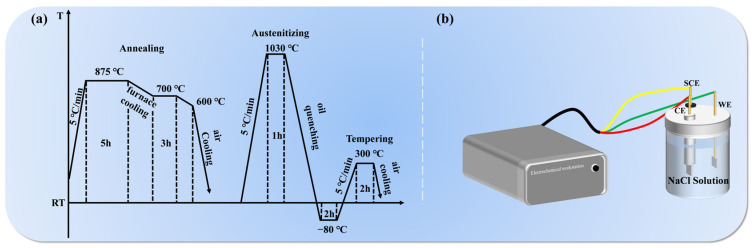
Schematic diagram of (**a**) heat treatment process, (**b**) electrochemical device of the experimental steels.

In this study, the N-free and 0.16 wt.% N MSS used was customized by a commercial company. The supply state is forged. Chemical analysis was performed using an optical emission spectrometer (ARL 4460, Thermo Scientific, Waltham, MA, USA), and to more accurately determine the nitrogen content, a special oxygen and nitrogen analyzer (ON736, LECO, Saint Joseph, MI, USA) was introduced for secondary detection. The chemical compositions are shown in Table 1. To make the description more concise, these two materials are designated as 0 N and 0.16 N in subsequent discussions. Spheroidal annealing treatment was carried out first during the material preparation process. The purpose was to uniform the structure, eliminate internal stress, and prepare the structure for subsequent quenching. The specific procedure was to place the sample in the furnace at 200 °C, then increase it to 875 °C and hold it for 5 h, then furnace cooled to 700 °C and kept for another 3 h, followed by cooling to 600 °C in furnace, finally air-cooled to room temperature [23]. After annealing, the samples were subjected to austenitization at 1030 °C for an hour. After austenitizing, the samples were quickly quenched in oil until cooled to ambient temperature. The quenched specimens underwent a low-temperature treatment at −80 °C for 2 h, followed by tempering at 300 °C for 2 h, illustrated in Figure 1a. The variation in phase fractions and precipitation with austenitizing temperature in the range of 500–1200 °C in the equilibrium conditions was calculated by the Thermo-Calc software 2023a equipped with the TCFE9 database.

### 2.2. Microstructure Characterization

Before the experiments, 10 × 10 × 3 mm samples were cut from heat-treated MSSs, grounded by 400–1500 grit abrasive paper in sequence, polished with 1.5 µm diamond spray, rinsed with deionized water and alcohol, and dried with hot air.

The microstructure was observed by scanning electron microscope (SEM, Zeiss Ultra-55, Jena, Germany) equipped with electron backscatter diffraction (EBSD, Oxford Instruments, Abingdon-on-Thames, UK) and transmission electron microscopy (TEM, FEI-Tecnai G2-F20, Thermo Fisher Scientific, Waltham, MA, USA).

SEM specimens etched using Vilella’s reagent (consisting of 1 g of picric acid, 10 mL of hydrochloric acid, and 100 mL of ethanol) [23], EBSD specimens were prepared by electro-polishing with 8 vol% perchloric acid and 92% ethanol at 30 V for 20 s. The EBSD tests, conducted at 20 KV with a step size of 0.16 μm, subsequently had their data analyzed using AztecCrystal 2.1. The TEM foils were ground to a thickness of 50 μm with a diameter of 3 mm and electro-polished via a twin-jet machine in a solution containing 8 vol% perchloric acid.

The element distribution of specimens was analyzed by the energy dispersive spectrometer (EDS) attached to SEM and an electron probe micro-analyzer (EPMA, JXA-8530 F, JEOL Co., Ltd., Kyoto, Japan). Phase characterizations were performed by X-ray diffraction (XRD, SmartLab 9 kW, Rigaku, Tokyo, Japan) with Cu Kα radiation, at a scanning rate of 2°/min from 20° to 90°.

### 2.3. Electrochemical Measurement

The specimens intended for electrochemical examination were first sliced into a shape measuring 10 × 10 × 3 mm. Subsequently, they were embedded in epoxy resin, thereby exposing approximately 0.38 cm^2^. Before the electrochemical testing, each specimen underwent the same grinding and polishing procedures as outlined in the sample preparation detailed in Section 2.2.

The electrochemical tests were performed using the Electrochemical workstation (CS235OH, Corrtest Instrument, Wuhan, China), which was equipped with a standard three-electrode cell (illustrated in Figure 1b). The tests included open-circuit potential (OCP) determination, electrochemical impedance spectroscopy (EIS) analysis, and potentiodynamic polarization (PDP) testing. In this system, the saturated calomel electrode (SCE) served as the reference electrode, a thin platinum sheet was used as the counter electrode (CE), and the sample to be measured was employed as the working electrode (WE). The electrolyte utilized in the experiment was 3.5 wt.% NaCl solution, prepared by dissolving analytically pure NaCl in deionized water and allowing it to stand until fully dissolved. At room temperature, OCP measurements were conducted for 2400 s for each sample. After a stable OCP value was reached, EIS tests were performed using an amplitude of ±10 mV, with a frequency sweep range from 10^−2^ Hz to 10^5^ Hz. The collected EIS data were thoroughly analyzed and fitted using ZSimpwin 3.60. The PDP tests were conducted at a scan rate of 0.3 mV/s, with a scanning potential range from −0.45V (vs. OCP) to 0.45V (vs. OCP). To ensure the reliability of the experimental data, all tests were conducted in triplicate.

## 3. Results and Discussion

### 3.1. Microstructural Characteristics

**Figure 2:**
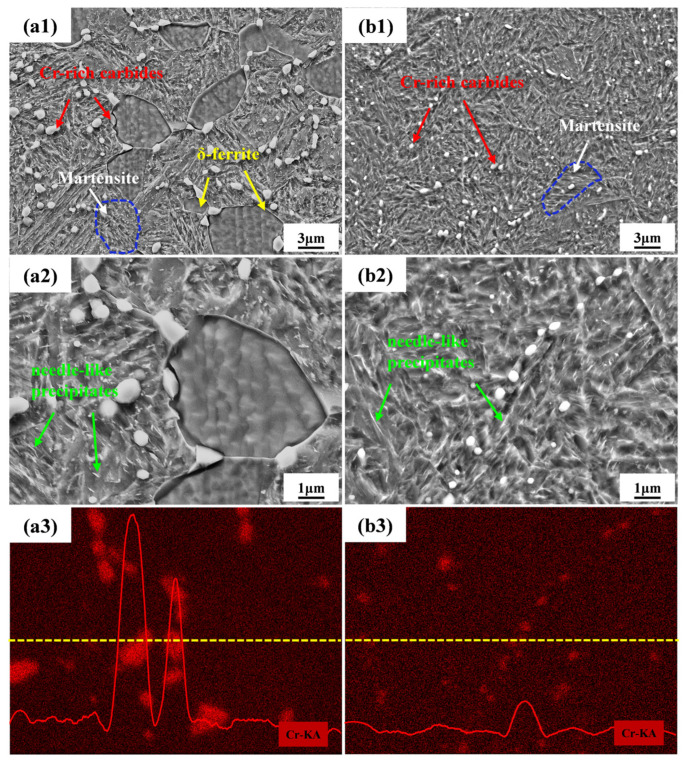
SEM micrographs and the EDS-mapping: (**a1**–**a3**) 0N and (**b1**–**b3**) 0.16 N specimens.

Figure 2 shows the microstructure of 0N and 0.16N specimens. In 0 N steel, reticulate δ-ferrite, martensite, and a large amount of coarse Cr-rich carbides were observed (Figure 2(a1,a3)). Moreover, needle-like precipitates were also found (Figure 2(a2)). However, the microstructure of the 0.16 N specimen consisted only of martensite without δ-ferrite, and fine Cr-rich carbides (Figure 2(b1,b3)). Compared to the 0 N specimen, the precipitates in the 0.16 N specimen were significantly refined, and the amount of precipitation was also decreased. Research has shown that the formation of the δ-ferrite in 0 N steel is mainly due to the high chromium equivalent and relatively low nickel equivalent. Nitrogen has a strong stabilizing effect on austenite. Adding nitrogen to martensitic stainless steel can effectively inhibit δ-ferrite [24]. It was observed that the precipitates were enriched with a significant amount of the Cr element as depicted in Figure 2(a3,b3). However, the 0.16 N specimen shows a more even distribution of Cr. Some researchers reported that in martensitic stainless steel, the addition of nitrogen can increase the state density of Fermi level, resulting in a short-range ordered arrangement, so that the distribution of Cr atoms is more uniform, which can inhibit the aggregation of Cr atoms, delaying the generation of Cr-rich precipitated phase, and reducing the size of precipitation [25].

**Figure 3:**
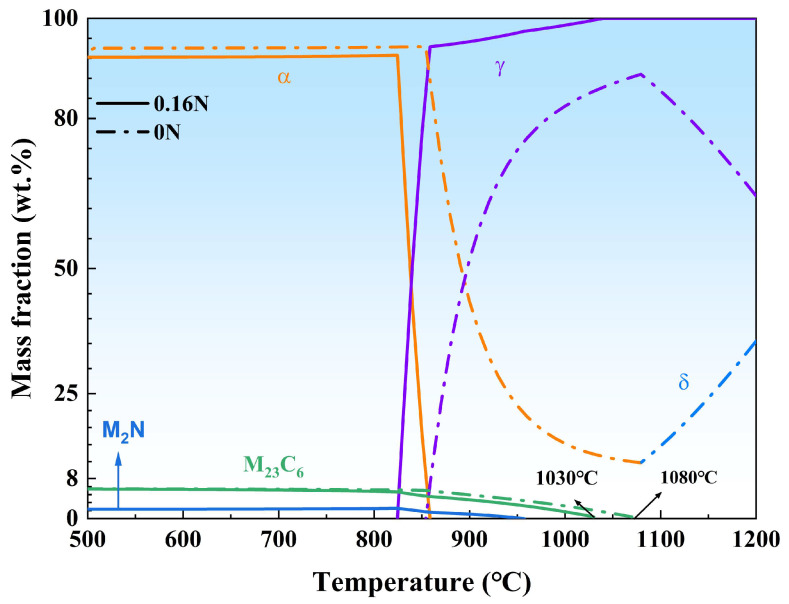
Variation of phase fractions in the temperature range calculated by Thermo-Calc software.

According to the calculation result of Thermo-Calc software based on the TCFE9 database, adding nitrogen significantly diminished the δ phase region, expanded the regions of M_2_N and γ phase, and reduced the dissolution temperature of M_23_C_6_ from 1080 °C to 1030 °C (Figure 3). The phase variation in SEM analysis agrees with this. The reason is that nitrogen in steel could impede the formation of M_23_C_6_ carbides and slow down the growth kinetics by diminishing the diffusion rate of Cr within the matrix [26,27,28], reducing the lattice parameter while increasing the interfacial mismatch [22,29,30,31].

**Figure 4:**
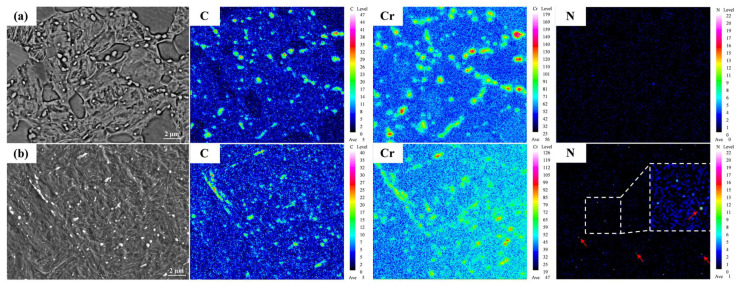
EPMA results of (**a**) 0 N and (**b**) 0.16 N MSSs. The red arrow is the N-rich area, and the white box is the magnification image of the N-rich area.

The distribution of elements in experimental steels is shown in Figure 4. The enrichment of chromium in the precipitates was clearly observed, which is in agreement with the EDS analysis results (Figure 2(a3,b3)). However, the distribution of nitrogen in the 0.16 N specimen was uniform. Wang et al. [32] reported in their study that replacing part of the carbon in an Fe-12.8%Cr-1.3%Ni-0.5%Mo-0.19%C welding wire with nitrogen effectively inhibited the formation of grain boundary precipitates and improved the distribution uniformity of elements in the material. Our observations are consistent with this finding.

**Figure 5:**
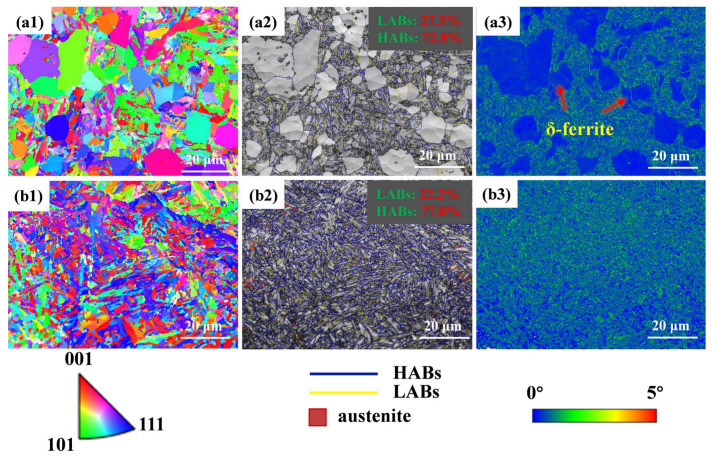
EBSD-inverse pole figure (IPF), phase, and KAM maps of (**a1**–**a3**) 0 N and (**b1**–**b3**) 0.16 N steels.

To investigate the microstructure characteristics of experimental steels in greater depth, an EBSD analysis was conducted. The color orientation diagrams of the inverse pole figure (IPF) of the experimental steels are shown in Figure 5(a1,b1), respectively. In the 0 N specimen, lath-shaped martensite and δ-ferrite, featuring blocky and bamboo-like morphologies, were observed, as indicated by the red arrows, and the average width of δ-ferrite was determined to be 5.75 ± 0.8 μm. To identify δ-ferrite more accurately, a Kernel Average Misorientation (KAM) analysis was carried out (as shown in Figure 5(a3,b3)). It is reported that the dislocation density of martensitic grains is proportional to its KAM value [33,34], that is, the larger the dislocation density, the higher the KAM value. On the contrary, δ-ferrite grains exhibit lower KAM values due to fewer dislocations.

As shown in Figure 5(a2), there is a small amount of austenite in the 0 N specimen. This is attributed to the reverse transformation of martensite to austenite during the tempering process, while the partitioning of C and Mn atoms into austenite improves its stability; therefore, austenite is retained at room temperature; Raabe et al. [35,36] reached this conclusion in a previous study. Compared with the 0 N sample, the austenite content in the 0.16 N sample (Figure 5(b2)) was significantly increased. This is because N significantly improves the stability of austenite, increasing the proportion of this phase in the sample of 0.16 N at room temperature [37]. Studies have shown that reversed austenite has a good promotion on pitting resistance because its presence enhances the stability of the passive film [38,39].

In the phase maps, the blue line represents high-angle grain boundaries (HABs, misorientation angle ≥ 15°) and the yellow one represents low-angle grain boundaries (LABs, 2° < misorientation angle < 15°). It is worth noting that with the addition of nitrogen, the density of martensitic lath increased significantly (Figure 5(b2)). In lath martensite, the lath boundaries are low-angle boundaries (LABs), while the packet and block boundaries generally appeared as high-angle boundaries (HABs), which was confirmed in a previous study [40,41].

**Figure 6:**
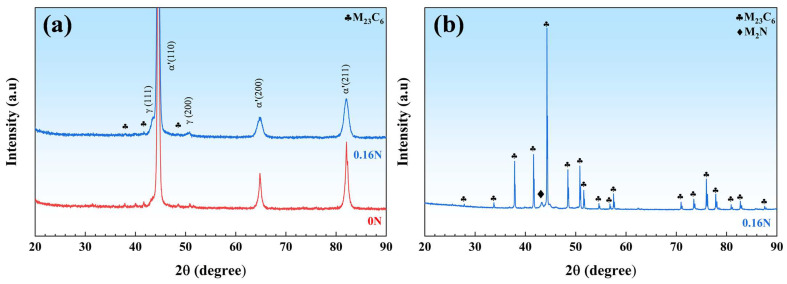
XRD patterns of (**a**) 0 N and 0.16 N MSSs, (**b**) electrolytically extracted precipitates in 0.16 N MSS.

An XRD analysis was performed to thoroughly investigate the types and the existing forms of precipitates. As shown in Figure 6a, the XRD spectra indicate that M_23_C_6_ carbides are the major precipitate. After adding nitrogen to the steel, there is a decrease in the amount of M_23_C_6_ carbides based on peak intensities. To further clarify the existence form of nitrogen in nitrogen-containing martensitic stainless bearing steel, Figure 6b shows the XRD pattern of electrolytically extracted precipitates. A peak representing M_2_N nitride appears.

**Figure 7:**
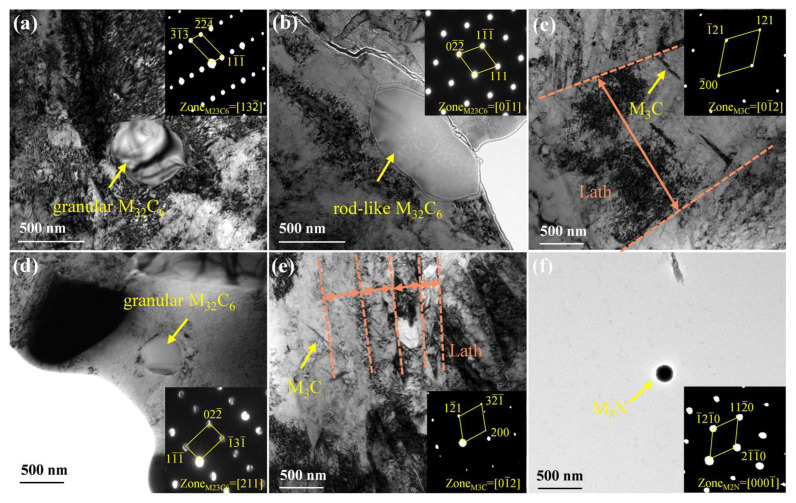
TEM images and SAED patterns of 0 N (**a**–**c**) and 0.16 N (**d**–**f**) MSSs.

Figure 7 shows the TEM micrographs and selected area electron diffraction (SAED) patterns of the precipitates in experimental steels. In N-free steel, granular, rod-like, and needle-like precipitates can be observed in Figure 7a–c, identified as M_23_C_6_ (~500 nm) and M_3_C carbides, respectively. Granular Cr-rich M_23_C_6_ (~300 nm), needle-like precipitates, and M_2_N (~120 nm) were observed in 0.16 N steel (Figure 7d–f). Therefore, combined with the above analysis results, it can be stated that nitrogen exists in 0.16N steel in solid solution and as Cr-rich nitrides. Furthermore, the width of martensitic laths also fluctuated simultaneously. More specifically, the average lath width decreased from 1.32 μm (Figure 7c) to 0.2 μm (Figure 7e). The width of the lath is closely related to the grain size of the prior austenite, and the width of the lath is narrowed after adding nitrogen, indicating that the grain is refined. The reason is that nitrogen, as an interstitial atom, migrates to the grain boundaries, and there is a strong interaction between Cr and N atoms, forming a Cr-N complex, which inhibits austenite grain boundary migration. At the same time, nitrogen and other elements form nitrogen-rich precipitates, which can pin the grain boundaries and inhibit the grain boundary migration, thus refining the prior austenite grains [20,42].

### 3.2. Effect of Nitrogen on Electrochemical Behavior

#### 3.2.1. OCP and Potentiodynamic Polarization Measurement

**Figure 8:**
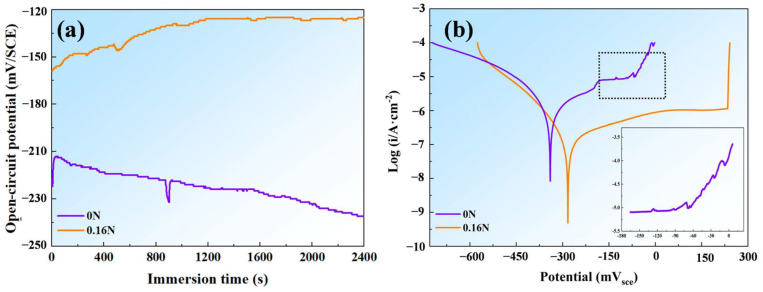
Results of electrochemical tests: (**a**) OCP, (**b**) PDP curves of the tested steels.

Figure 8a features the OCP measurements. With the increase in experimental time, the OCP of 0.16 N steel remained stable (around −160 mV). In contrast, nitrogen-free steel exhibited potential transient fluctuations, which may have resulted from the metastable pitting nucleation and subsequent recovery [43], as well as a consistent decrease, reaching a much lower value.

Figure 8b shows the PDP curves of the experimental MSSs, and the pitting potential (E_pit_) of the test steels was determined when the current density at the specimen surface exceeded 0.1 mA/cm^2^ (listed in Table 2). From the curves, the 0.16 N specimen exhibits a clear passive region before the applied potentials reach E_pit_. However, in the 0 N steel, in the passive region, severe current fluctuations can be seen, due to the existence of massive Cr-rich precipitates, signifying the extensive initiation and subsequent repassivation of metastable pits [44]. These observations indicate that the inclusion of 0.16 wt.% N can notably suppress the occurrence of metastable pitting and substantially elevate the E_pit_ value.

Table 2 gives the electrochemical parameters obtained from the potentiodynamic polarization curves. The corrosion potential (E_corr_) and corrosion current density (i_corr_) were obtained via the extrapolation method. It is noted that the OCP values of the samples (Figure 8a) are larger than their E_corr_ values (Figure 8b). This may be due to the formation of a passivation film on the surface of the specimen during their long-term immersion in the solution during the OCP measurement, and the obtained potential value should be the potential of the oxide film [45]. Thus, the E_corr_ measured for the sample in this work should correspond to the potential of the bare metal surface in its active condition, which should be lower than the potential of the passivation film [46]. According to the PDP test methodology, i_pass_ is measured when the polarization potential reaches the passive region. The passivation current density (i_pass_) of the 0.16 N sample is 1.26 μA/cm^2^, lower than that of the 0 N sample (8.32 μA/cm^2^), which demonstrates that the passive film of the nitrogen-containing MSS is more protective when exposed to a chloride environment.

#### 3.2.2. Electrochemical Impedance Spectroscopy Measurements

**Figure 9:**
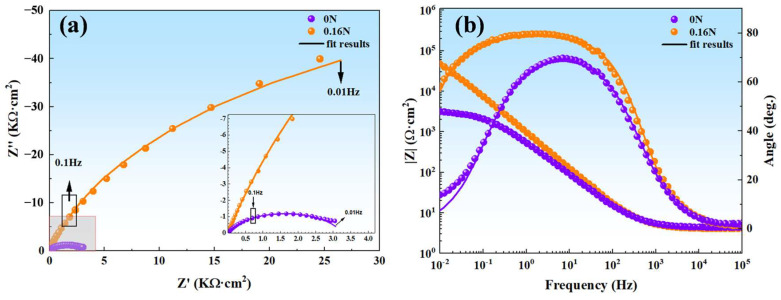
EIS results of 0 N and 0.16 N MSSs: (**a**) Nyquist plots and (**b**) Bode plots.

To delve deeper into understanding how nitrogen addition affects the corrosion process, we conducted EIS tests. As illustrated in the Nyquist plot (Figure 9a), the radius (R) of the capacitive arc for nitrogen-containing MSS is significantly larger than that of nitrogen-free MSS. The corrosion resistance of steel is intimately correlated with the radius (R) of the capacitive arc [47]. The larger radius indicates that the more difficult the charge transfer, the greater the polarized resistance and the better the corrosion resistance [48,49].

Figure 9b shows the Bode plots of the experimental MSSs. In the amplitude plot, |Z|_0.01Hz_ is the impedance mode value at a fixed frequency of 0.01 Hz, which corresponds to the polarization resistance. It is commonly used to evaluate the barrier characteristics of the corrosion layer on the material surface, reflecting the corrosion resistance of the material in the solution [50]. The impedance modulus at low frequency (|Z|_0.01Hz_) of 0 N steel is nearly 10^4^ Ω·cm^2^, almost one order of magnitude lower than |Z|_0.01Hz_ of 0.16 N steel (10^5^ Ω·cm^2^).

In the phase angle diagram, the characteristics of the phase angle change curve can provide important information about the corrosion tendency of the material. The width of the phase angle curve and the height of the phase angle peak are the key indices to evaluate the corrosion resistance of materials. From the phase angle diagram (Figure 9b), the maximum phase angle value of the 0.16 N sample is close to 82°, while the 0 N sample is approximately 76°. Meanwhile, the 0.16 N steel has a wider frequency range of phase angles, which indicates that a more stable passivation film is formed on the surface of the 0.16 N specimen in 3.5% NaCl solution. The reason is that the high phase angle peak usually leads to the formation of a more stable and dense passivation film or protective layer on the material’s surface, which can effectively isolate the contact between the corrosive medium and the material matrix. The higher the peak phase angle, the more stable the sample surface is and the stronger the corrosion resistance. Secondly, a wider frequency range of phase angles usually means that the material can maintain its stability over a wider frequency range, which means it can resist corrosion under different environments and conditions [51,52,53].

**Figure 10:**
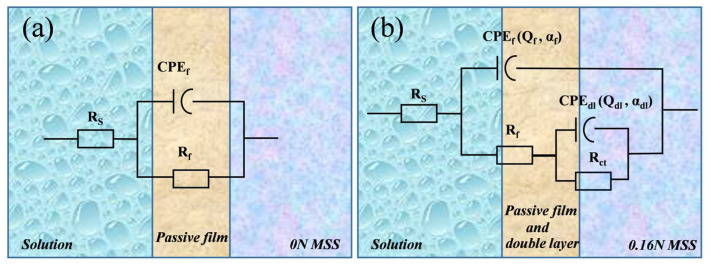
Equivalent circuit used to fit EIS data: (**a**) 0 N model, (**b**) 0.16 N model.

The equivalent circuits for analyzing the impedance data from the experiment are illustrated in Figure 10. The EIS data were fitted and analyzed using the Z-SimpWin3.60 software. From the Bode plots (Figure 9b), the 0 N sample exhibits one time constant, whereas the diagrams for the 0.16 N sample display two time constants, namely the double layer and the passive film [54]. The equivalent circuits with one time constant (Figure 10a) and two time constants (Figure 10b) were applied to fit the obtained EIS data.

As shown in Figure 10, the critical electrochemical parameters, including solution resistance (R_s_), passive film resistance (R_f_), constant phase element of passive film (CPE_f_), charge transfer resistance (R_ct_), and constant phase element for double layer (CPE_dl_) were estimated (summarized in Table 3).

The impedance of the CPE (Z(CPE)) can be described by the following equation [55]:

(1) $$Z(CPE)=Q^{-1}{\left(j\omega \right)}^{-\alpha }$$

where *Q* is the admittance value of CPE, *j* is an imaginary number (*j*^2^ = 1), ω is the angular frequency, and *α_f_* is the dispersion coefficient [56], where *α_f_* ranges from −1 to 1. When α_f_ = 1, the CPE becomes a pure capacitor. However, due to the dispersion effect, it usually deviates from the pure capacitance, so αf decreases [57].

Table 3 lists the best fitting parameters for EIS. The goodness of fit between experimental data and simulation results was quantitatively evaluated using the chi-square (χ^2^) method [57]. The calculated χ^2^ values of the 0 N and 0.16 N samples were 9.3 × 10^−4^ and 1.5 × 10^−4^, respectively, implying a good quality of fitting. The dispersion coefficient of the α_f_ values for all the samples is smaller than 1, suggesting that the impedance behavior deviates from that of a pure capacitor and is attributed to the inhomogeneities of the passive film [58,59]. The α_f_ value of 0.16 N is closer to 1, indicating a more uniform distribution of the passive film formed on the martensitic stainless steel with added nitrogen. In addition, it is generally acknowledged that the rate of the interfacial reactions can be determined by the polarization resistance (R_p_), defined as R_p_ = (Z_F_)_ω=0_ (Z_F_ represents the faradic impedance and “ω” is the angular frequency, respectively), which is directly related to the corrosion resistance of metal [60]. Therefore, the R_p_ of equivalent circuits in Figure 10a,b can be calculated as R_p_ = R_f_ and R_p_ = R_f_ + R_ct_, respectively [61]. As is shown in Table 3, the 0.16 N sample exhibits the highest R_p_ value of 1.24 × 10^2^ kΩ·cm^2^. This means that after adding nitrogen to MSS, the performance and ability of the passive film have been enhanced.

#### 3.2.3. Corrosion Mechanism Analysis

For 0 N and 0.16 N martensitic stainless steels, precipitates have a significant impact on their corrosion performance. It is believed that Cr-rich M_23_C_6_ carbides give rise to Cr-depleted zones in their vicinity, which subsequently hinder the development of protective passivation films and elevate the vulnerability to pitting [60,62]. The addition of N to martensitic stainless steel inhibits the precipitation of M_23_C_6_, alleviates the consumption of Cr, reduces the Cr depletion to a certain extent, and ultimately improves the pitting resistance of the steel.

**Figure 11:**
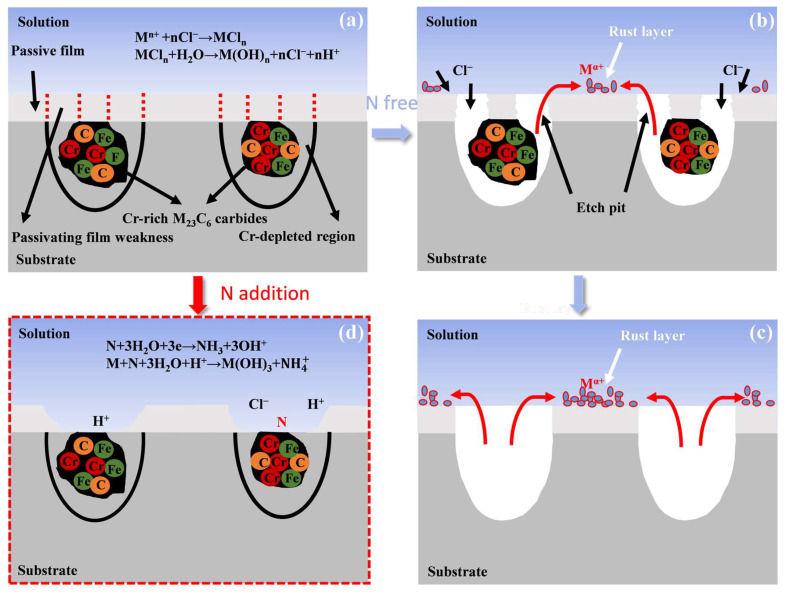
Schematic illustration of the effect of nitrogen and Cr-rich M_23_C_6_ carbides on the pitting behavior of the experimental MSSs. (**a**) formation of passive film on the surface, (**b**,**c**) pitting mechanism of the 0N steel, (**d**) pitting mechanism of the 0.16N steel.

To further illustrate the pitting mechanism, a schematic diagram was inferred based on existing theories, as shown in Figure 11. The surface of stainless steel forms a passivation film, providing protection against electrolyte corrosion. The passive layer in the vicinity of the carbides displays microscopic chemical discontinuities [61], resulting in areas depleted of Cr, as illustrated in Figure 11a. This leads to the formation of a weakly passivated film that allows chloride ions to permeate easily and become a preferred site for pit nucleation in chlorine solutions. Research has revealed that chloride ions can react with the metal substrate at the interface between the metal and the film, forming metal chlorides. In solutions containing chloride ions, the failure (or breakdown) of the passivation layer is rooted in the gradual accumulation of chloride ions at the metal/film interface after they penetrate through the protective film layer [18]. In the initial stage of pitting, due to the destruction of the passivation film, Cl^−^ accelerates the erosion of the matrix, and the metal cation (M^+^) in the matrix dissolves and diffuses, forming a rust layer around the pits [63,64] (Figure 11b). During the pitting process, the cationic hydration product H^+^ dissolved in the pit is formed. When the metal dissolution rate is accelerated, the hydration process is also significantly accelerated, resulting in a gradual decrease in the surface pH value of the pit. When the pH value in the pit is below a certain critical value, it will hinder the repassivation process of the passivation film, which will promote the deterioration of the pit and eventually form a stable pitting (Figure 11c).

However, according to the adsorption mechanism, the adsorption of Cl^−^ on the passivation film leads to the formation of MCl_x_ (M stands for Fe, Cr, etc.) and the rupture of the passivation film [65,66]. With the thinning of the passivation film, the presence of nitride in it can induce the repulsion and desorption of Cl^−^, thus inhibiting the corrosion caused by chloride ions [65,67]. In addition, nitrogen is enriched at the metal/oxide film interface and reacts with H^+^ to form NH_4_^+^ or NH_3_, which plays a key role in buffering the pH value in the metastable pit, facilitates the early repassivation process, inhibits the development of the growth pit, and, thus, enhances the pitting resistance (Figure 11d).

## 4. Conclusions

The present study delves into the profound influence of nitrogen on the precipitates, phase evolution, and corrosion properties, especially pitting corrosion resistance in martensitic stainless steel. The following primary conclusions are drawn from this study:

(1) In this study, the nitrogen in martensitic stainless steel mainly exists in Cr-rich nitride and solid solutions. Nitrogen addition effectively inhibits the production of δ-ferrite. The precipitates are all rich in Cr, and the Cr distribution in 0.16 N steel is more uniform, which can reduce the adverse effect of Cr depletion on corrosion resistance.

(2) Nitrogen addition significantly reduces the current fluctuations during polarization tests, that is, reduces the sensitivity of metastable pitting, enlarges the passivation zone, and makes the passivation film more uniform and stable. At the same time, the pitting potential (E_pit_) increased significantly (241.69 mV), which is two orders of magnitude higher than the 0 N specimen, and the passive current density (i_pass_) decreased, but the change is not significant.

(3) The electrochemical impedance spectroscopy (EIS) results confirm that the 0.16 N steel impedance modulus at low frequency (|Z|_0.01 Hz_) and capacitive loop radius are larger, and the frequency range of phase angle curve is wider, i.e., the passive film of the nitrogen-containing MSS is more protective when exposed to a Cl^-^-containing environment.

## Figures and Tables

**Table 1:** Chemical compositions of the experimental MSSs (wt.%).

Steels	C	Cr	Mo	N	Mn	Si	Fe
0N	0.30	15.17	1.02	/	0.45	0.55	Bal.
0.16N	0.31	15.16	0.97	0.16	0.42	0.51	Bal.

**Table 2:** The electrochemical parameters of experimental MSSs derived from OCP and PDP measurements.

Samples	OCP/mV	i_pass_/(μA/cm^2^)	E_pit_/mV	i_corr_/(μA/cm^2^)	E_corr_/mV
0 N	−250.2	8.3	−6.8	0.9	−339.5
0.16 N	−124.7	1.2	241.6	0.1	−281.0

**Table 3:** The optimal fitting results of EIS parameters obtained through Z-SimpWin.

Samples	R_s_ (Ω·cm^2^)	CPE_f_		R_f_ (kΩ·cm^2^)	CPE_dl_		R_ct_ (kΩ·cm^2^)	Χ^2^
		Q_f_ (MΩ^−1^/cm^−2^s^α^)	α_f_		Q_dl_ (MΩ^−1^/cm^−2^s^α^)	α_dl_		
0 N	4.86	4.77 × 10^−4^	0.80	4.05	/	/	/	9.31 × 10^−4^
0.16 N	4.05	8.39 × 10^−5^	0.95	7.27 × 10^−3^	1.19 × 10^−4^	0.82	1.24 × 10^2^	1.53 × 10^−4^

## Data Availability

The datasets used and/or analyzed during the current study will be made available from the corresponding author upon reasonable request.
